# The American Academy of Microbiology discusses gain-of-function research of concern (GOFROC) and enhanced potential pandemic pathogens (ePPP)

**DOI:** 10.1128/mbio.02761-23

**Published:** 2023-12-11

**Authors:** Arturo Casadevall, Michael J. Imperiale, Nguyen K. Nguyen, Vanessa Sperandio

**Affiliations:** 1 Former Chair of the American Academy of Microbiology Committee of Governors, Department of Molecular Microbiology and Immunology, Johns Hopkins School of Public Health, Baltimore, Maryland, USA; 2 Chair of Steering Committee of Workshop on Impact Assessment of Research on Infectious Agents, Department of Microbiology and Immunology, University of Michigan, Ann Arbor, Michigan, USA; 3 Director of the American Academy of Microbiology, American Society for Microbiology, Washington, USA; 4 Chair of the American Academy of Microbiology Committee of Governors, Department of Medical Microbiology and Immunology, University of Wisconsin, Madison, Wisconsin, USA

## Abstract

The American Academy of Microbiology convened a workshop bringing together scientists with varied opinions on the conduct of gain-of-function research of concern (GOFROC) and enhanced pathogen with pandemic potential (ePPP) research. Five findings were: (1) research on infectious agents is necessary for understanding, monitoring, and developing treatments and prevention measures against these agents; (2) gain-of-function research of concern or ePPP research makes up a very small fraction of all biological research; (3) clearly defined terminologies for research of concern should be developed by the scientific community to avoid public confusion and highlight its practical benefits; (4) harmonized biorisk management standardization, training, mentoring, and reporting can help improve safety and security for laboratory workers and the public; and (5) expanded engagement and collaboration of scientists with policymakers and the public, including increased transparency on the risks and rewards of research with infectious agents, is needed.

## EDITORIAL

The realization that biomedical research could be a source of new and more lethal infectious diseases took hold around the turn of the 21^st^ century. The mailing of five envelopes containing anthrax spores in the days after the terrorist attacks of September 11, 2001, combined frequent infectious disease outbreaks that have culminated in the ongoing COVID-19 pandemic have greatly heightened awareness of microbial threats. In the early 2000s a series of scientific publications reporting a poxvirus that evaded immunity (1), the vulnerability of the milk distribution system to botulinum toxin (2) and the design of complement evading poxvirus (3) provided examples of how biomedical research could generate information that was potentially applicable for nefarious uses, including the generation of pathogens with increased virulence. In 2012 two groups reported that avian influenza virus could be adapted for mammalian transmissibility (4, 5), an event that produced a crisis in the field of infectious research by providing a clear example of how laboratory manipulation could create a new virus with pandemic potential. Some viewed such experiments as too dangerous to conduct and others argued that such experiments provided critically important information for preparedness against pandemic threats.

The controversy was never resolved, and the debate was re-ignited in the early 2020s as humanity faced the COVID-19 pandemic which brought to the fore concerns about viral engineering, laboratory accidents, and the untoward potential consequences of infectious disease research (6). This heightened awareness has led to greater scrutiny of, increased concern about, additional regulation of, and even proposed legislation governing research on pathogens with pandemic potential. In 2023, amid the increasing politicization of infectious disease research, the American Academy of Microbiology (Academy), a scientific think-tank at ASM, convened a workshop bringing together a diverse set of scientists with varied opinions on the conduct of gain-of-function research of concern (GOFROC) and enhanced pathogen of pandemic potential (ePPP) research. On 13 September 2023, a report on this workshop was published (https://asm.org/Reports/Impact-Assessment-of-Research-on-Infectious-Agents) (7). The workshop produced five key insights:

1. Research on infectious agents is necessary for understanding, monitoring, and developing treatments and prevention measures against these agents. Moreover, basic research provides knowledge and insights that may prove useful in the future in ways unknown at the present. For example, basic coronavirus and mRNA vaccine research starting in the 1980s and 1990s enabled the rapid development of vaccines for COVID-19 in the 2020s.
2. Gain-of-function research of concern or ePPP research makes up a very small fraction of all biological research. However, this category of experiments raises legitimate concerns about biosafety and biosecurity risks.
3. Clearly defined terminologies for research of concern should be developed by the scientific community to avoid public confusion and highlight its practical benefits.
4. Harmonized biorisk management standardization, training, mentoring, and reporting can help improve safety and security for laboratory workers and the public.
5. Expanded engagement and collaboration of scientists with policymakers and the public, including increased transparency on the risks and rewards of research with infectious agents, is needed.

The workshop successfully provided a venue for communication between advocates and opponents of gain-of-function research of concern and found areas of consensus, as stated in the above five findings. One of the participants noted that everyone agreed on more than 90% of the issues discussed with the remaining 10% providing differences that were the source of the controversy. At the heart of the controversy is a disagreement about the risks and benefits of such research. Since the risks are usually estimated and the benefits of research often cannot be anticipated, the differences in opinion reflect how these are weighed by individuals and their personal values. One accomplishment of the workshop report is that it captured all the arguments made for and against such research in one document, which to our knowledge has not been accomplished previously. We are hopeful by listing arguments for or against such research that the report increases understanding of the complex issues involved and leads to further discussions that perhaps can identify the best way forward.

Going forward, as a think-tank at ASM, the Academy will strive to serve as a trusted broker in future discussions and use its good offices to help maintain open lines of communication among the scientists and provide credible scientific information to the microbiology community, policy makers, and interested parties. Even when differences are very difficult to bridge, scientific organizations like the ASM can play important roles in helping find the way forward to the benefit of science and humanity. Furthermore, scientists need to remain informed about the nature of the controversy and we are hopeful that recommendations 4 and 5 will help build public confidence in research activities.
